# Vaccine literacy and attitudes toward ımmunization among adults in primary care

**DOI:** 10.1590/1806-9282.20252237

**Published:** 2026-07-10

**Authors:** Birgül Deniz Doğan, Murat Doğan

**Affiliations:** 1 Ahi Evran University, Faculty of Medicine, Department of Family Medicine – Kırşehir, Türkiye.

**Keywords:** Immunization, Vaccination, Health knowledge, Attitudes, practice, Health literacy, Adult

## Abstract

**OBJECTIVE:**

Vaccine hesitancy continues to be a significant global public health issue, and our understanding of how vaccine literacy influences immunization attitudes is incomplete. The aim of this study was to evaluate vaccine literacy and attitudes toward immunization among adults in Central Anatolia, Turkey, and to examine the association between vaccine literacy dimensions and immunization attitudes.

**METHODS:**

A cross-sectional study was conducted between February and May 2025 among 928 adults attending Family Health Centers in Kırşehir. Data were collected through face-to-face interviews using a structured sociodemographic questionnaire, the Health Literacy about Vaccination in Adults Scale, and the Attitudes Toward Immunization Scale. Nonparametric tests, Spearman correlation analyses, and multiple linear regression were applied.

**RESULTS:**

Participants showed moderate levels across all vaccine literacy domains and moderately positive immunization attitudes. Correlations between Health Literacy about Vaccination in Adults Scale subscales and Attitudes Toward Immunization Scale scores were generally weak. Communicative vaccine literacy showed a weak but important negative relationship with the overall Attitudes Toward Immunization Scale score (r=-0.077, p=0.018) and the belief/opinion part (r=-0.121). In regression analysis, none of the vaccine literacy subscales significantly predicted immunization attitudes, and the model explained only 0.4% of the variance (Adjusted R^2^=0.004, p=0.090).

**CONCLUSION:**

Vaccine literacy alone has limited explanatory value for immunization attitudes. These findings indicate that attitudes are shaped by factors beyond cognitive literacy and suggest that interventions should extend beyond information provision.

## INTRODUCTION

Vaccination is one of the most effective public health interventions for reducing morbidity and mortality from infectious diseases^1^. In addition, vaccines developed against oncogenic viruses such as human papillomavirus (HPV) and hepatitis B virus (HBV) also play an important role in reducing the burden of infection-related cancers^2^. Despite this, vaccine hesitancy has become a major public health issue on a global scale in recent years^3^. In particular, the insufficient coverage of vaccination in the adult population has increased the importance of this issue.

During the Coronavirus Disease 2019 (COVID-19) pandemic, misinformation spread through digital platforms, infodemics, and weakened institutional trust emerged as important factors determining vaccination behavior^4^. Vaccine hesitancy is a complex phenomenon influenced by multidimensional factors such as misinformation, trust in the healthcare system, social media exposure, and individual values^5^.

These developments suggest that simply increasing access to information may not be sufficient. In this context, vaccine literacy is defined as an important cognitive resource influencing vaccination behavior^6^. However, growing evidence suggests that information alone may not be sufficient to explain vaccine hesitancy or vaccination behavior^7,8^.

The literature shows that attitudes are influenced not only by the level of knowledge but also by contextual factors such as trust, previous experiences, and the perceived reliability of the information source^3,5^. Nevertheless, knowledge-related constructs remain a central component in theoretical models of vaccination decision-making. Based on this framework, we hypothesized that higher vaccine literacy would be associated with more positive attitudes toward immunization in adults. This hypothesis was empirically tested in the present study.

Studies examining the relationship between vaccine literacy and vaccination attitudes in adult primary care populations remain limited. Therefore, this study aimed to evaluate the association between vaccine literacy and attitudes toward immunization using the Health Literacy about Vaccination in Adults Scale (HLVa-IT) and the Attitudes Toward Immunization Scale (AITO) among adults living in Kırşehir.

## METHODS

A cross-sectional descriptive study was carried out between February and May 2025 in Family Health Centers serving the city center of Kırşehir, Central Anatolia, Turkey. Adults aged 18 years and older who were registered at these centers constituted the study population. Overall, 928 individuals who fulfilled the inclusion criteria and provided informed consent were enrolled.

Sample size estimation was performed using Epi Info™ version 7.2, assuming a 95% confidence level, a 5% margin of error, and an effect size of 0.5, which indicated a minimum requirement of 385 participants. To enhance statistical power, a larger sample was included. Participants were recruited through simple random sampling.

Data collection was conducted via face-to-face interviews using a structured sociodemographic form together with validated measurement instruments. Collected variables comprised age, sex, educational attainment, occupation, income status, place of residence, and the presence of chronic disease. Monthly household income was classified in relation to the national minimum wage as below, equal to, or above the minimum wage.

Vaccine literacy was evaluated using HLVa-IT, which was originally developed by Biasio et al. and subsequently adapted into Turkish by Demir and Koçoğlu-Tanyer^9,10^. The HLVa-IT comprises 14 items grouped into three domains: Functional, Communicative/Interactive, and Critical Vaccine Literacy. Functional items are reverse-coded, with higher scores reflecting greater vaccine literacy. In the Turkish validation, confirmatory factor analysis demonstrated good model fit (Comparative Fit Index [CFI]=0.958, Root Mean Square Error of Approximation [RMSEA]=0.067), and Cronbach's alpha coefficients were 0.90, 0.82, and 0.81 for the respective subscales.

To enhance clarity, representative examples of scale items are provided. For functional vaccine literacy, an illustrative item assesses the participant's ability to understand written vaccine information (e.g., "How easy is it for you to understand written information about vaccines?"). Communicative/interactive literacy is reflected by items addressing information-seeking and discussion behaviors (e.g., "How often do you discuss vaccine-related information with healthcare professionals?"). Critical vaccine literacy includes items evaluating appraisal of information sources (e.g., "How confident are you in judging whether vaccine information from different sources is reliable?").

Attitudes toward immunization were assessed using AITO, originally developed by Cvjetkovic et al. and validated in Turkish by Özümit and Sarı^11,12^. The scale consists of 14 items rated on a five-point Likert scale and includes two subscales: Attitude and Belief/Opinion. Negatively phrased items were reverse-coded, and higher scores indicated more favorable attitudes toward immunization. Cronbach's alpha values for the subscales were 0.87 and 0.90.

Illustrative items from the ATTO include statements reflecting general acceptance of vaccines (e.g., "Vaccines are effective in preventing serious diseases") and beliefs or concerns about immunization (e.g., "I am concerned about possible side effects of vaccines"). Participants rated their agreement with each statement on a five-point Likert scale.

### Ethics committee approval

The study was performed in accordance with the principles of the Declaration of Helsinki. Ethical approval was granted by the Clinical Research Ethics Committee of Kırşehir Ahi Evran University Faculty of Medicine (Decision No: 2025-06/70, February 2025). Written informed consent was obtained from all participants prior to data collection.

### Statistical analysis

Statistical analyses were carried out using International Business Machines Corporation Statistical Package for the Social Sciences Statistics version 25.0. Data distribution was evaluated with the Shapiro-Wilk test, and non-parametric methods were applied accordingly. Continuous variables are presented as median (Q1–Q3), whereas categorical variables are summarized as frequencies and percentages. Group comparisons were performed using the Mann-Whitney U or Kruskal-Wallis tests with Bonferroni-adjusted post-hoc analyses. Associations between variables were examined using Spearman's rank correlation coefficient. Multivariable linear regression analysis was used to explore whether vaccine literacy subscales predicted attitudes toward immunization. A p<0.05 was considered statistically significant.

## RESULTS

The mean age of the participants was 49.38±15.48 years, with a median age of 53 years (Q1–Q3: 37–60). The sociodemographic characteristics of the study population are presented in Table 1.

**Table 1:** Sociodemographic characteristics of the participants (n=928).

Variables	Category	n (%)
Age, median (Q1–Q3)	53.0 (37–60)	–
Gender	Male	470 (50.6)
	Women	458 (49.4)
Educational background	Illiterate	37 (4)
	Primary school	275 (29.6)
	Secondary school	232 (25.0)
	High school	312 (33.6)
	University degree	70 (7.5)
	Postgraduate degree	2 (0.2)
Occupation	Civil servant	305 (32.9)
	Farmer	276 (29.7)
	Self-employed/tradesperson	187 (20.2)
	Homemaker	149 (16.1)
	Not working	11 (1.2)
Place of residence	Urban (city center)	505 (54.4)
	Town	252 (27.2)
	Village	171 (18.4)
Income level	Low	283 (30.5)
	Moderate	600 (64.7)
	High	45 (4.8)
Primary source for health problems	Internet resources	162 (17.5)
	Health institutions	537 (57.9)
	Friends/family	126 (13.6)
	Other	103 (11.1)

Median scores indicated moderate levels of vaccine literacy across all HLVa-IT subscales and moderately positive attitudes toward immunization. Internal consistency coefficients ranged from acceptable to good for both HLVa-IT and AITO subscales (Cronbach's α between 0.718 and 0.85) (Table 2).

**Table 2:** Descriptive statistics of vaccine literacy and immunization attitude scales and their correlations.

(A) Descriptive statistics and reliability			
Subscale	Median (Q1–Q3)	Cronbach's alpha	Classification
Functional vaccine literacy	2.60 (2.00–3.00)	0.718	Acceptable
Communicative vaccine literacy	2.60 (2.00–3.00)	0.812	Good
Critical vaccine literacy	2.50 (2.00–3.00)	0.785	Acceptable
Attitude subscale	2.88 (2.50–3.13)	0.85	Good
Belief/opinion subscale	2.83 (2.50–3.33)	0.83	Good
AITO total score	40.00 (37.00–44.00)	0.775	Acceptable
**(B) Correlations between HLVa-IT subscales and immunization attitudes (Spearman's rho)**			
**HLVa-IT subscales**	**Attitude subscale r_s_ (p)**	**Belief/opinion r_b_ (p)**	**AITO total r_b_ (p)**
Functional vaccine literacy	-0.034 (0.301)	**0.083 (0.011)**	0.028 (0.396)
Communicative vaccine literacy	-0.021 (0.527)	**-0.121 (<0.001)**	**-0.077 (0.018)**
Critical vaccine literacy	-0.019 (0.570)	-0.060 (0.069)	0.028 (0.265)

Values represent Spearman's rho (r_b_) and p-values. Bold values indicate statistically significant correlations (p<0.05). HLVa-IT: Health Literacy about Vaccination in Adults; AITO: Attitudes Toward Immunization Scale; r_b_: Spearman correlation coefficient.

No statistically significant differences were observed between men and women in any HLVa-IT subscale or in AITO scores (p>0.05).

Spearman correlation analysis revealed generally weak associations between HLVa-IT subscales and immunization attitudes (Table 2). Functional Vaccine Literacy showed a weak positive correlation with the AITO Belief/Opinion subscale (r=0.083, p=0.011), whereas Communicative Vaccine Literacy demonstrated weak negative correlations with both the Belief/Opinion subscale (r=-0.121, p<0.001) and the AITO total score (r=-0.077, p=0.018). No statistically significant correlations were observed between Critical Vaccine Literacy and AITO scores.

Multiple linear regression analysis demonstrated that none of the HLVa-IT subscales significantly predicted attitudes toward immunization (Table 3). The overall regression model was not statistically significant [F(3, 924)=2.174, p=0.090] and explained only a very small proportion of the variance in AITO total scores (R^2^=0.007; adjusted R^2^=0.004). No evidence of multicollinearity was detected (Variance Inflation Factor [VIF] values<2.1).

**Table 3:** Multiple linear regression of Health Literacy about Vaccination in Adults Scale subscales predicting Attitudes Toward Immunization Scale total scores.

Predictors	B	SE	β	t	p	Tolerance	VIF
Constant	41.675	1.306	–	31.921	<0.001	–	–
Functional VL	0.139	0.335	0.014	0.415	0.678	0.957	1.045
Communicative VL	-0.767	0.420	-0.084	-1.825	0.068	0.503	1.987
Critical VL	0.052	0.399	0.006	0.129	0.897	0.498	2.007

Note: B: unstandardized coefficient; SE: standard error; β: standardized coefficient; t: t-value; p: p-value; VIF: variance inflation factor; VL: vaccine literacy. Model summary: R=0.084, R^2^=0.007, Adjusted R^2^=0.004, F(3, 924)=2.174, p=0.090. Dependent variable: AITO total score (Attitudes Toward Immunization Scale).

## DISCUSSION

In this study, the vaccine literacy levels and attitudes toward vaccination among adults in Central Anatolia were assessed using the HLVa-IT and AITO scales. Our aim was to empirically test the effect of vaccine literacy on attitudes toward immunization. However, contrary to our expectations, the analysis results revealed a rather weak relationship. The fact that the regression analysis model could explain only 0.4% of the variance (Adj. R^2^=0.004) suggests that attitudes toward vaccination are explained to a limited extent by vaccine literacy dimensions and that attitudes may not be explained solely by vaccine literacy dimensions, with other factors also playing a role. While the HLVa-IT scale measures individuals’ capacity to access, understand, and evaluate vaccine-related information, it is understood that attitudinal orientations are not determined solely by cognitive competence. The literature emphasizes that more complex psychosocial processes, such as risk perception, social norms, identity, past experiences, and emotional evaluations, may contribute to this construct^13,14^. Therefore, our expectation that higher levels of vaccine-related knowledge would be reflected in more positive attitudes was not supported by the findings.

The "knowledge gap model," which has been used for many years to explain health behaviors, assumes that individuals will adopt the correct health behavior when they have sufficient information^3,7^. However, this model has been shown to be limited, especially in the field of vaccines^15^. An editorial published in The Lancet Child and Adolescent Health emphasizes that vaccine hesitancy poses a global risk and that this problem cannot be solved by information campaigns alone^15^. Similarly, studies conducted among parent populations report that vaccine refusal can persist even when the level of knowledge is sufficient^16,17^. Although the safety and efficacy of vaccines have been clearly approved by the World Health Organization's Global Advisory Committee on Vaccine Safety, vaccine hesitancy is reported in the vast majority of countries worldwide^1,18^. Even among individuals with high awareness of the disease's mortality during the COVID-19 pandemic, reluctance to vaccinate has been observed^19^.

These findings suggest that attitudes involve emotional, social, and cultural components to an extent that cannot be explained solely by cognitive information processing. Correlation analyses have shown no significant relationship between functional or critical vaccine literacy and general vaccination attitudes.

In contrast, a weak but statistically significant negative correlation was found between communicative vaccine literacy and AITO total scores (r=-0.077, p=0.018) and belief/opinion subscale scores (r=-0.121, p<0.001). This finding suggests that showing more interest in vaccine-related information may not always be associated with more positive attitudes. Particularly in digital information environments, individuals’ tendency to seek out content that aligns with their existing views (confirmation bias) may be a potential mechanism influencing this relationship^20^. In this context, communication literacy skills may support the search for information consistent with existing attitudes in some individuals. The literature reports that individuals who are hesitant about vaccines are more active in seeking information online and may be more receptive to anti-vaccine content^21^. Therefore, future interventions may need to target not only information-seeking skills but also the capacity to evaluate the quality and reliability of the information obtained.

In our study, 57.9% of participants indicated healthcare institutions as their primary source of information on health-related issues, demonstrating that healthcare institutions remain the most frequently consulted source for health-related information. This finding supports the central role of healthcare professionals in vaccine communication. Indeed, the literature shows that healthcare workers’ recommendations have a strong effect on vaccination behavior^22^. Furthermore, it is emphasized that clear, empathetic, and culturally sensitive communication reinforces trust and supports vaccine acceptance^23^. In this context, strategies that prioritize trust-building and contextual communication may be more effective than approaches based solely on information transfer in healthcare settings.

Similarly, in Brazil, multisectoral collaborations with the education sector in HPV vaccination programs have increased vaccination rates, suggesting that vaccine attitudes are related not only to individual knowledge levels but also to social and institutional contexts. Furthermore, Australia's school-based national HPV vaccination program has achieved high coverage rates and produced strong projections for the elimination of cervical cancer^24,25^. Therefore, strategies to improve vaccine attitudes should go beyond a narrow focus on information transfer; they should include multidimensional approaches that strengthen reliable information channels, support empathetic and culturally sensitive relationships between healthcare professionals and patients, and consider the social context.

This study has several limitations. First, its cross-sectional design precludes causal inferences between vaccine literacy and attitudes. Second, attitudes were assessed, but actual vaccination behavior was not measured. Third, key psychosocial variables such as trust, risk perception, and social norms were not directly evaluated, which may partly explain the low explanatory power of the regression model. Finally, as the study was conducted in a single province, generalizability to other sociocultural contexts may be limited.

## CONCLUSION

Contrary to our initial hypothesis, vaccine literacy was not meaningfully associated with attitudes toward immunization. The explanatory power of literacy dimensions was negligible. These findings suggest that increasing vaccine literacy alone is unlikely to substantially influence immunization attitudes in adult primary care populations.

Future research should move beyond literacy-focused models and directly investigate psychosocial determinants such as trust, perceived risk, social norms, and identity-based factors. Understanding these broader mechanisms may provide a more accurate framework for interpreting vaccination attitudes.

## Data Availability

The datasets generated and/or analyzed during the current study are available from the corresponding author upon reasonable request.
